# A Self-Directed Mobile Intervention (WaznApp) to Promote Weight Control Among Employees at a Lebanese University: Protocol for a Feasibility Pilot Randomized Controlled Trial

**DOI:** 10.2196/resprot.9793

**Published:** 2018-05-16

**Authors:** Marco Bardus, Ghassan Hamadeh, Bouchra Hayek, Rawan Al Kherfan

**Affiliations:** ^1^ Department of Health Promotion and Community Health Faculty of Health Sciences American University of Beirut Beirut Lebanon; ^2^ Department of Family Medicine Faculty of Medicine American University of Beirut Beirut Lebanon

**Keywords:** mobile apps, weight loss, physical activity, healthy diet, workplace, mHealth, randomized controlled trial

## Abstract

**Background:**

Overweight and obesity have become major health problems globally with more than 1.9 billion overweight adults. In Lebanon, the prevalence of obesity and overweight is 65.4% combined. Risk factors of obesity and overweight are preventable and can be addressed by modifications in the environment and in an individual’s lifestyle. Mobile technologies are increasingly used in behavioral, self-directed weight management interventions, providing users with additional opportunities to attain weight control (weight loss, weight gain prevention, etc). Mobile apps may allow for the delivery of Just-in-Time Adaptive Interventions (JITAIs), which provide support through skill building, emotional support, and instrumental support, following the participants’ progress. A few commercially available apps offer JITAI features, but no studies have tested their efficacy.

**Objective:**

The primary objective of this study is to examine the feasibility of a self-directed weight loss intervention, targeting employees of an academic institution, using a virtual coaching app with JITAI features (Lark) and a self-help calorie-counting app (MyFitnessPal). The secondary objective is to estimate the effects of the intervention on main study outcomes.

**Methods:**

This study is a single-center, parallel, randomized controlled trial with 2 study arms (intervention and control). Participants will be randomly allocated in equal proportions to the intervention (Lark) and control groups (MyFitnessPal). To be eligible for this study, participants must be employed full- or part-time at the university or its medical center, able to read English, have a smartphone, and be interested in controlling their weight. Recruitment strategies entail email invitations, printed posters, and social media postings. We will assess quantitative rates of recruitment, adherence, and retention, self-reported app quality using the user version of the Mobile App Rating Scale. We will also assess changes in weight-related outcomes (absolute weight and waist circumference), behavioral outcomes (physical activity and diet), and cognitive factors (motivation to participate in the trial and to manage weight).

**Results:**

WaznApp was funded in June 2017, and recruitment started in March 2018.

**Conclusions:**

This study will provide information as to whether the selected mobile apps offer a feasible solution for promoting weight management in an academic workplace. The results will inform a larger trial whose results might be replicated in similar workplaces in Lebanon and the Middle East and North Africa region, and will be used as a benchmark for further investigations in other settings and similar target groups.

**Trial Registration:**

ClinicalTrials.gov NCT03321331; https://clinicaltrials.gov/ct2/show/NCT03321331 (Archived by WebCite at http://www.webcitation.org/6ys9NOLo5)

**Registered Report Identifier:**

RR1-10.2196/9793

## Introduction

### Background

Noncommunicable diseases (NCDs), also known as chronic diseases, are one of the major global public health challenges of the 21st century, being responsible for about 40 million deaths per year, 15 of which are premature (ie, between 30 and 69 years) [1]. The majority of these deaths occur in low- and middle-income countries (LMICs). According to the World Health Organization’s (WHO’s) global report, cardiovascular diseases, cancer, diabetes, and chronic respiratory diseases are responsible for 82% of all NCD deaths [2]. In Lebanon, the prevalence of NCDs accounts for 85% of all deaths. NCDs are the product of 4 main risk factors: tobacco use, physical inactivity, the harmful use of alcohol, and unhealthy diets, which, in turn, lead to 4 key metabolic changes (raised blood pressure, overweight and obesity, raised blood glucose, and raised cholesterol) [1]. With regard to overweight and obesity, globally, in 2014, there were more than 1.9 billion overweight adults, representing 39% of the world population. Of these, 600 million were obese [3]. In Lebanon, the prevalence of obesity and overweight is 65.4% (obesity accounting for 27.4% and overweight for 38%) [4]. A fundamental cause of obesity and overweight is an energy imbalance between caloric intake and caloric consumption. This imbalance is due to global trends of increased availability and intake of energy-dense foods that are high in sugar and saturated fats, and insufficient physical activity, due to the sedentary nature of many forms of work, modes of transportation, and increased urbanization [3]. All these behavioral risk factors are preventable, as they can be addressed by modifications in the environment and lifestyle of individuals (ie, increasing physical activity, reducing sedentary time, and following a healthy diet) [5].

The effectiveness of nonsurgical and behavioral weight management interventions has been demonstrated in several systematic reviews and meta-analyses [6,7]. For example, commercially available weight loss treatments, such as WeightWatchers, and pharmaceutical products, such as Qsymia, were found to be cost-effective strategies to achieve weight loss [8]. Effective behavioral interventions should include both physical activity and dietary components to reach larger and sustained effects [9]. Self-monitoring is one of the most effective change techniques [10] included in behavioral weight loss interventions, as evidence shows that people who report weight monitoring on a daily or weekly basis tend to be more successful in attaining weight loss goals [11,12]. Self-monitoring improves the awareness of caloric and food intake, increases self-efficacy, and permits the evaluation of any change or progress over time [13]. However, long-term weight loss and maintenance interventions that are delivered face-to-face usually require substantial work of a specialist workforce and sizeable resources, both from the participants and the service providers.

In the past decade, “health services and information delivered or enhanced through the internet and related technologies” (ie, “eHealth”) [14] have transformed the way patients interact with the health care system [15] and engage with their own health [16]. eHealth technologies allow users to monitor, track, and inform their health; communicate between health agencies and external stakeholders in terms of health; and collect, manage, and use health data [17]. In this context, new strategies for behavioral weight management interventions have been developed [18-22], with the aim to provide users with the support necessary to attain weight loss and, at the same time, contain costs [23]. The potential of eHealth and mobile health (mHealth) is especially relevant to LMICs, where phone ownership is rising rapidly, but access to health care services is often limited. Recent systematic reviews on eHealth behavioral interventions addressing NCDs and their risk factors in LMICs show promising results [24,25]. The attention toward mHealth apps is also justified by the high penetration rates of these technologies. According to a 2014 Pew Research Center survey, in Lebanon, the penetration rate of mobile phones was 86%, with smartphones reaching 45%, the highest penetration rate in the Middle East and North Africa (MENA) region [26]. Smartphone ownership is as high as 60% among adults aged 18 to 29 years and 55% among adults aged 30 to 49 years [26]. More recent data (October 2016 to November 2017) available from Net Marketshare show that mobile and tablet represent 61% of the market in Lebanon, with Android capturing 61% and iOS capturing 38% of the Lebanese market.

Mobile phones, and particularly apps, have been considered convenient intervention platforms as they are portable, appealing, and universal [27]. Mobile phones can be used for self-directed interventions for weight management. Self-directed interventions are those that “require minimal professional contact (eg, provision of initial instructions) or no professional contact and can be easily used with existing infrastructure and in the context of users’ everyday lives” [28]. Mobile apps started to show some suggestive evidence of effectiveness [18,29], with studies reporting positive effects in weight reduction when apps were employed as a supplement to telephone coaching [30]. However, small, nonsignificant effects were reported when apps were used as a standalone tool [31,32]. Smartphones are the ideal platform for delivering self-directed, Just-in-Time Adaptive Interventions (JITAIs), which are treatment programs that, as the name suggests, adapt to the patients’ progress, eg, when they attain goals or positively respond to treatment [33]. JITAIs can provide support through skill building (coping, making decisions, planning, etc), emotional support (encouragement, etc), and instrumental support (feedback, etc) when users need these features the most [33,34]. JITAIs are complex, algorithm-dependent interventions based on several design principles, which include decision points and outcomes that would inform the provision of tailored feedback (ie, tailoring variables), intervention options (based on *if-then* conditions grounded on behavioral theories, aimed at addressing short-term behavioral outcomes), and decision rules. For a more detailed overview of the JITAI framework, we refer the reader to the seminar report by Nahum-Shani et al [34]. Mobile devices are adequate platforms for delivering feasible and scalable JITAIs, given the smartphone technology advancements that are providing users with continuous monitoring and personalized coping strategies [34]. Nevertheless, little is known about the efficacy of mobile apps acting as main components of JITAIs for weight management.

According to the WHO’s global action plan for the prevention and control of NCDs [35], workplaces are one of the most important settings for health promotion as they are the gateway to a large number of people (about 65% of the world’s population is employed) [36]. In the last two decades, many public health efforts have been made, and numerous interventions have been conducted to tackle these problems. There is a wide evidence supporting the effectiveness of workplace interventions for the prevention of obesity [37-40], and many national governments adopted policy decisions for promoting health through workplaces. In response to the WHO call for action, the Lebanese Ministry of Public Health released a plan for the prevention and control of NCDs in 2016 [4], which provides a set of guidelines for action, despite excluding workplaces as a setting for priority interventions. This gap is being filled by the American University of Beirut (AUB) and its Medical Center (AUBMC) [41], which have expressed their commitment toward building a healthier campus and community through a long-term strategic plan endorsed in the Health 2025 vision.

### Aims of This Study

The overarching goal of this study is to determine the feasibility and preliminary efficacy of a self-directed weight management intervention, targeting university employees and delivered entirely through mobile apps. Specifically, the study aims to (1) evaluate the acceptability and feasibility of using 2 commercially available mHealth apps for weight management; (2) evaluate the feasibility of implementation strategies, retention, and adherence to the study, fidelity to the protocol, assessments, and data collection procedures; and (3) estimate the effects of the interventions on weight-related outcomes (eg, weight, body mass index, and waist circumference), as well as on behavioral (physical activity and diet), and cognitive factors related to weight loss (motivation to lose weight, to engage in physical activity, and follow a healthy diet).

## Methods

### Participants and Procedures

Participants are employees working at AUB and its Medical Center (AUBMC), located in Beirut, Lebanon. Employees include faculty (n=1182), of which 41.03% (485/1182) are female; nonacademic staff at AUB (n=1266) of which 46.99% (595/1266) are female; and AUBMC staff (n=3038) of which 52.00% (1580/3038) are female [41]. The employer did not influence the design of the study or its methodology. The recruitment strategy does not rely on the endorsement of any AUB or AUBMC authorities. Participants will be informed orally and through written consent forms that their participation is completely voluntary, free of charge, and no penalty will be pressed for those not willing to participate.

To be enrolled in the study, participants must (1) be employed full- or part-time; (2) be able to read, write, and understand English; (3) own a smartphone with either Android (v4.4 or above) or iOS (v8 or later); and (4) be interested in better controlling their weight (ie, losing weight, preventing weight gain, maintaining weight lost, or gaining weight in a healthy way). Exclusion criteria include the following: (1) being full-time students who cannot prove their status as full- or part-time employees at AUB or AUBMC; (2) not being able to read, write, and understand English; (3) not owning a smartphone with either Android (v4.4 or above) or iOS (v8 or later); (4) having physical disabilities preventing from exercising or walking; (5) being on a special diet for treatment of chronic conditions (eg, diabetes); (6) being diagnosed with anorexia or bulimia nervosa; (7) being on weight loss medications; and (8) having undergone bariatric surgery in the past 3 months. The trial is registered at ClinicalTrials.gov [NCT03321331]. The protocol of this study has been approved by the local Institutional Review Board (ref #FHS.MB.07/SBS-2017-0416).

### Recruitment and Randomization

Participants will be recruited through email invitations sent from the AUB Health and Wellness Center, and printed posters hanged on billboards on campus. A digital version of the poster with a shortened link (bit.ly/WaznApp) and QR-code linking to an eligibility screener survey will be displayed on monitors on campus TV screens. Invitations to enroll will also be circulated on social media using the research team’s personal and professional social networks (Facebook, Twitter, LinkedIn, and ResearchGate). Emails, posters, and social media postings will contain a link leading to an eligibility screener survey, which will first display an informed consent form. The survey, designed using LimeSurvey, is hosted on AUB servers. At the end of the form, employees will be instructed to complete their enrolment by visiting the University Health Services (UHS) clinics, where nurses will verify their eligibility and take basic anthropometric measurements (ie, height, weight, and waist circumference). Nurses will fill out paper-based forms, thereby cross-checking their eligibility to participate in the study. If participants are eligible, nurses will inform the participants that the research team will contact them via email with information about the next steps in the study. Nurses will provide a hard copy of the consent form to all participants.

Randomization procedures will take place after employees are confirmed to be enrolled in the study. A random sequence based on the minimization procedure will be generated using a computer program, following Altman and Bland’s approach [42]. The minimization procedure allows to balance the allocation of study participants to a prespecified number of treatment groups as soon as they enroll in the study, considering participant characteristics (ie, stratification by gender, age, and anthropometric features) collected during the eligibility phase. The program used to perform the minimization procedure is QMinim [43]. A statistician from the Faculty of Health Sciences will generate the sequence using the program. The random sequence will be uploaded to the REDCap (Research Electronic Data Capture) platform [44]. The research team will inform participants about their allocation after the visit, with a welcome email.

As we need to provide intervention participants with a link to download a full version of the app, allocation concealment and blinding cannot be applied. However, efforts will be implemented to reduce allocation bias. First, the clinical staff weighing participants at baseline and Week 12 will be blinded to treatment groups. Second, after the baseline clinic visit and the initial account set-up, minimization procedures will be undertaken by a statistician, not involving the principal investigator. Third, the intervention is completely delivered via mobile apps. The only contact is related to study procedures and data collection. Finally, data analysis will be conducted on masked data.

### WaznApp Study Overview

The WaznApp study is part of a larger project entitled “Can commercial mobile apps for weight management be used in interventions? Bridging the gap between usability, theoretical adherence, and user experience.” The project included a formative phase, which was based on a user-centered heuristic evaluation study, aimed at understanding how members of the employee community perceive 6 weight management apps (Lark, MyFitnessPal, SparkPeople, MyPlate, My Diet Diary, and My Diet Coach). The 6 apps were selected because they achieved the highest total scores on the Mobile App Rating Scale (MARS) [45], in a recent expert review [46]. In the heuristic evaluation study, 36 employees were randomly assigned to use one of these apps for 2 weeks. At the end of this period, they submitted their app quality evaluation using the user version of the MARS scale (uMARS) [47]. Three apps achieved the highest mean ratings for the total app quality score (Lark=4.0; MyPlate=3.8; MyFitnessPal=3.7). Lark clearly emerged as the app with the highest quality scores. In addition, this app has been recently employed in an observational study, involving 70 diabetic patients, who lost 2.4% of their weight at baseline after about 15 weeks [48]. Users of MyPlate reported several functionality issues, and its database is not as complete and responsive as its popular counterpart MyFitnessPal, which is one of the most downloaded apps for dietary tracking [49]. Compared with another popular app (Lose It!), MyFitnessPal allows users to automatically track activity through the phone or through integrations with many wearable devices [50]. For these reasons, we selected Lark and MyFitnessPal as trial apps.

The WaznApp study is a 12-week, prospective, single-center, parallel, randomized controlled trial with 2 study arms. The intervention arm will use Lark, a mobile coach, which provides interactive counseling through a chat-style interface by delivering motivational feedback, goal setting, and emotional social support. Lark acts as an adaptive intervention [33] and provides feedback on several outcomes (see, eg, the text and app-based Cell Phone Intervention for You trial [51,52]). The content of the messages depends on the way users interact with the app. The content is based on several behavior change techniques, such as goal setting, reviewing behavioral goals, feedback, and social support [46]. The users input their activities, foods or weight, but the app tracks automatically their active time using proprietary algorithms and phone motion sensors. The control group will use MyFitnessPal, a calorie-counting app that does not include JITAI components and relies on the user input for food tracking. MyFitnessPal acts as the control condition, as it provides limited social support, which is present in Lark, and it is an important feature that is generally lacking in calorie-counting apps [53].

### Description of Treatment Arms

#### Intervention Arm (Just-in-Time Adaptive Intervention)

**Figure 1:**
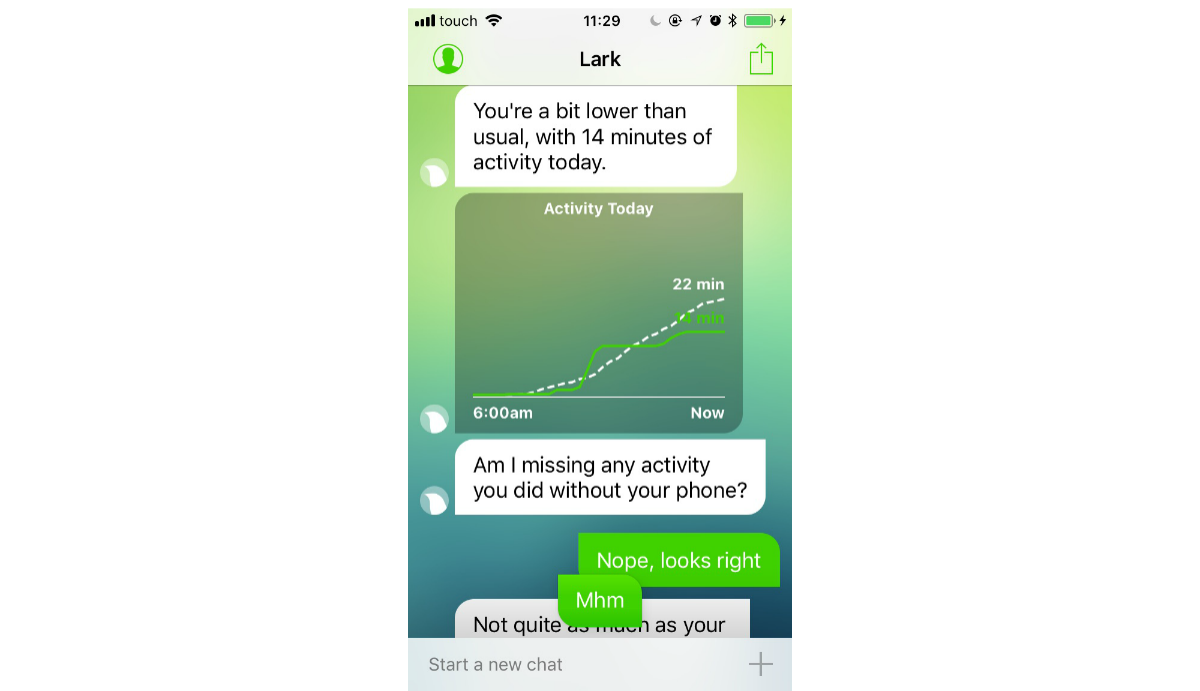
Example of self-monitoring of physical activity and visual feedback on behavior.

**Figure 2:**
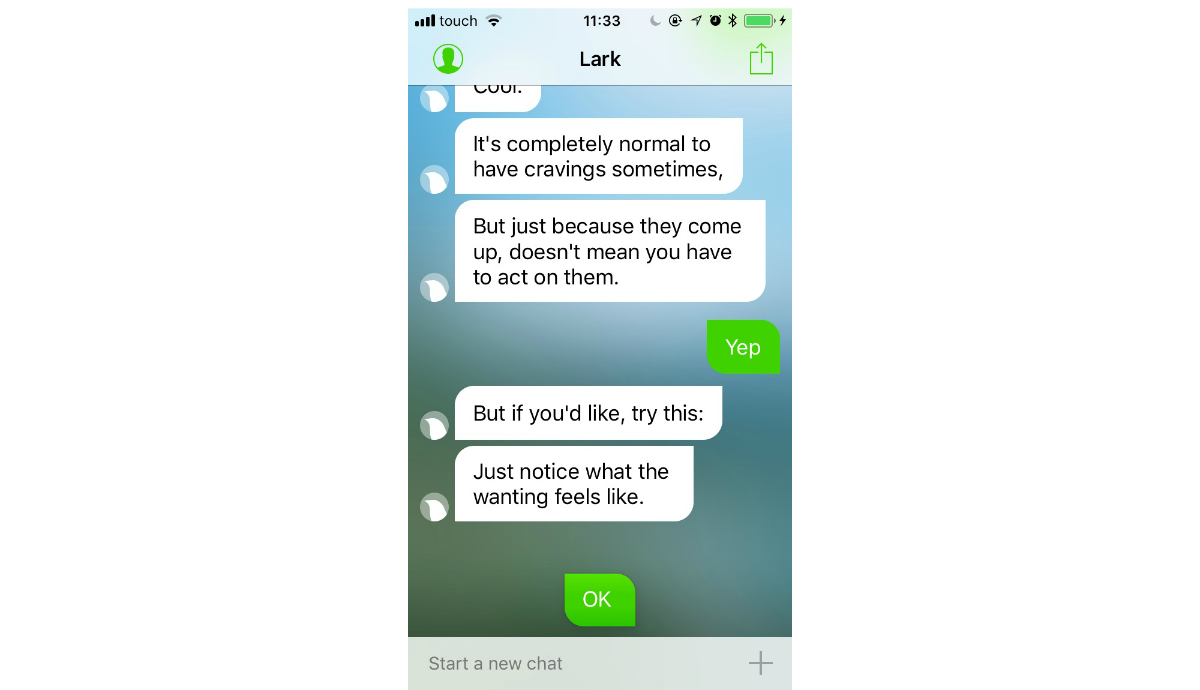
Example of mindful logging with problem-solving skills.

**Figure 3:**
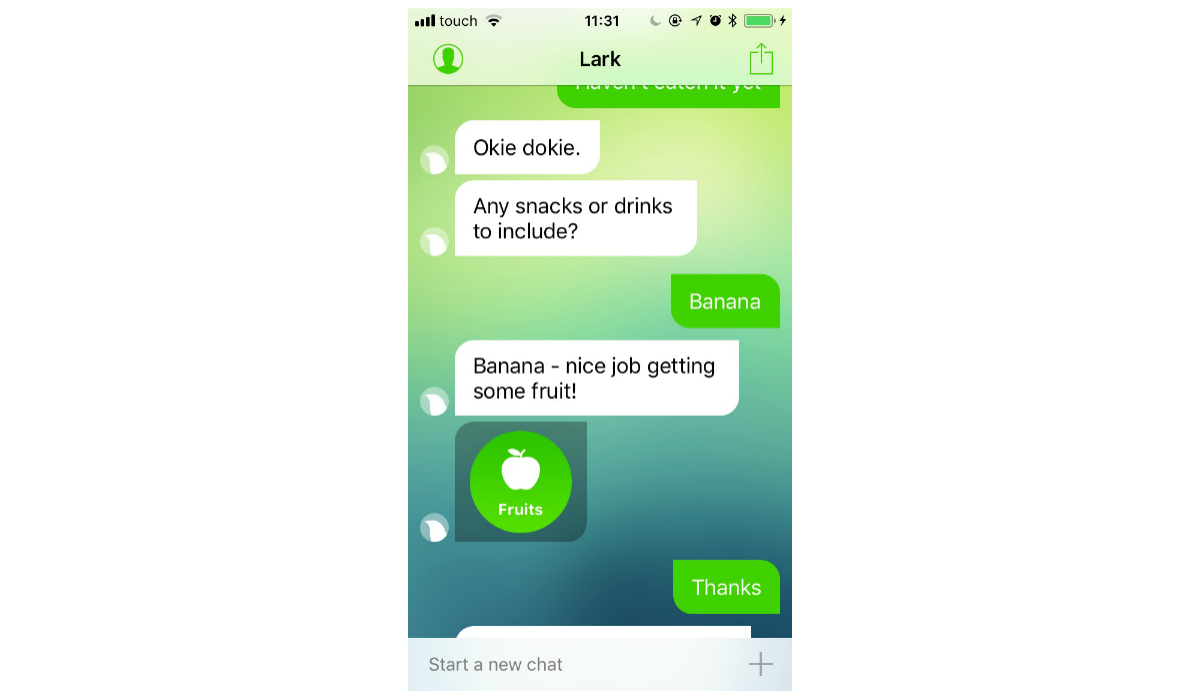
Example of positive reinforcement (ie, “social reward”).

Participants in the intervention arm will use Lark Pro for 12 weeks. Lark, developed by Lark Technologies Ltd, is a personal weight management health coach based on artificial intelligence that interacts with the user in a chat-like format. Lark Pro includes a personalized health plan and nutrition coaching, as well as physical activity, weight, sleep, and mood tracking. Once the app is launched, Lark checks the data that the user inputted (ie, food meals; physical activities such as walks, runs, bike, and workouts; and weight) or that was automatically logged through the phone motion sensors (ie, activity, sleep) or external devices (eg, Apple Watch, digital scales). On the basis of this information, Lark generates interactive conversations every time individuals use the app. Conversations about meal logging are geared toward food choices and portions rather than calorie counting; conversations may include messages that provide solutions to problematic situations, fostering action planning and problem-solving skills. The app sends notifications to prompt the users to review their activity throughout the day. Following the behavior change technique taxonomy v1 [10], Lark includes the following techniques: “goal setting” of behavior and outcomes (eg, activity, weight), “review outcome goals,” “self-monitoring” of behavior and outcomes, and “feedback” on behavior or on outcomes of behavior (Figure 1). In addition, users may receive “information about health consequences” (eg, when inputting a “muffin” as a meal, the app may respond: “That wasn’t the healthiest of meals/When I say this I am referring to the quality of the actual foods rather than taking into account the quantity you eat, but obviously eating an entire dessert is less healthy than having a single taste”). Lark also helps users to develop “problem-solving” skills (Figure 2), providing alternative solutions (“What’s a healthier choice?” response: “You could try fruit, which is sweet, filling, and packed with antioxidants”). The app also provides “emotional support” (eg, “know that when it comes to weight loss, ups and downs are typical”) and “positive reinforcement” (Figure 3). Sometimes, the app suggests information based on “credible sources,” as it has been developed in collaboration with researchers from Stanford and Harvard universities.

In agreement with Lark Technologies, users will utilize an app that will not be updated until the end of the study, to minimize variability in the intervention delivery.

#### Control Arm (No Just-in-Time Adaptive Intervention)

Participants in the control arm will be assigned to use MyFitnessPal. Similar to participants in the JITAI arm, they will be instructed to use the app for 12 weeks. MyFitnessPal does not include JITAI components, but it allows users to keep track of their caloric intake and energy expenditure. According to the previously cited review of mHealth apps for weight management [46], MyFitnessPal also has features that can be associated with effective behavior change techniques, including self-monitoring of behavior and outcomes, goal setting, and feedback (similar to Lark). In MyFitnessPal, social support is limited to comments and “likes” from friends of its restricted user community; hence, it might include “social comparisons” and “social reward” behavior change techniques [10]. MyFitnessPal acts as an active control arm as it relies almost exclusively on self-control, compared with Lark, as the latter provides an emotional social support from the coach, who provides positive enforcement and motivational messages to further encourage behavior change.

#### Additional Components

This study will be delivered almost entirely through mobile phones. Face-to-face interactions with the research team will be kept at minimum and used only to ensure that the mobile apps have been installed correctly, and there are no issues related to the use of technology during the intervention period. Participants will be instructed via manuals and tutorials delivered via email on how to download and use the apps, complete the surveys, and how to use the food composition tables for use in the Middle East [54], available from the local library. Manuals for completing dietary assessment using the Web-based Automated Self-Administered 24-hour (ASA24) Dietary Assessment Tool, version 2016, are available directly on the tool’s website. Emails will be used to communicate with study participants and to send them links for completing Web-based intermediate and follow-up assessments (collected through REDCap). The research team will encourage participants to keep their phone with them to track steps and active time, as the phones automatically collect the number of steps walked using built-in accelerometers.

#### Incentives for Participation

Study participants will not receive any payment for their participation, but they will be awarded “WaznApp karma points” (WAKpts) for completing tasks related to the study. Karma points are a nonmonetary measure of contribution to the study [55] and are awarded when participants in both intervention groups duly fill the Web-based questionnaires and food records. Each question filled in is worth 1 WAKpt. The points are according to the number of tasks completed. If the participants collect at least 600 points, they will enter the final lottery for Fitbit products, which will take place at the end of the study.

#### Intervention Administration and Fidelity

The intervention is delivered by the apps in an automated way. Neither the research team nor the developers have direct contact with participants and will not prompt app use. The research team will send emails to participants only to remind them about data collection procedures via REDCap. All research assistants have undertaken Collaborative Institutional Training Initiative training and acquired certification. Nurses collecting data and eligibility information will be trained before the start of the study. The research team will meet the nurses and provide instructions on how to fill the eligibility forms. Intervention fidelity will be monitored weekly, through meetings with research assistants and through the support of a project management platform (Basecamp), which keeps track of activities and tasks. Participants’ compliance with the intervention will be assessed through the email data collection points at baseline and Weeks 4, 8, and 12.

### Outcomes

In this study, primary outcomes are those related to the feasibility, whereas secondary outcomes are those related to the preliminary efficacy. The timeline for data collection is specified for each outcome below. We will collect data mostly through mobile-friendly, Web-based surveys. Participants will be invited to fill the questionnaires via email, which will contain personalized links to the surveys. Before the start of the study, all instruments will be pilot tested.

#### Feasibility and Acceptability Outcomes

As done in other similar trial [56], feasibility measures include quantitative rates of recruitment, adherence, and retention. Adherence to the study will be based on the number of data collection points completed and on qualitative feedback related to the study requirements. For Lark users (intervention group), we will use the logs of the app about quality (ie, detail and accuracy) and quantity of meals logged, daily activities logged, weight, sleep duration, and the number of conversations with the Lark coach as measures of intervention adherence. Retention rates will be calculated at the end of the study based on the number of participants who successfully complete the study, excluding dropouts. For both intervention and control groups, we will ask participants to provide information about their weekly app usage (hours/week). In Week 4, 8, and 12 surveys, we will add instructions on how to find the information on iPhones, using the built-in battery saving analytics, or Android phones, using the free app called *Frequency: App Usage Tracking*.

Acceptability of the trial will be assessed using qualitative feedback collected at each data point through open-ended questions (ie, “What are your major concerns about the study procedures, if you have any?”; “What are your major concerns about the app you have used?”; “What are your major concerns about the questionnaires?” Please elaborate your answers in the box below). Satisfaction with the program will be assessed through a 7-point rating scale (semantic differential), ranging from extremely satisfied to extremely dissatisfied. An open-ended question will give participants the option to elaborate on their response. These measures are calculated at the end of the study (Week 12). Satisfaction with the app will be assessed through the uMARS [47]. The uMARS scale provides a measure of app quality based on the average of 4 subdomains: engagement, functionality, aesthetics, and information; it also includes the subjective quality domain. Each of the subdomains is based on the average value of multiple items, assessed through 5-point Likert scales (engagement: 5 items; functionality and information: 4 items; aesthetics: 3 items; and subjective quality: 4 items). In the uMARS developmental study [47], the total scale and subscales achieved good and excellent internal consistency: engagement (Cronbach alpha=.80); functionality (alpha=.70); aesthetics (alpha=.71); information (alpha=.78), total (alpha=.90), and subjective quality (alpha=.78). As the uMARS tool requires that users utilize the app before rating it, app quality will be collected at Weeks 4, 8, and 12 (to allow for test-retest reliability estimates).

#### Secondary Outcomes

Secondary outcome measures include changes in weight-related outcomes (absolute weight and waist circumference), behavioral outcomes (physical activity and diet), and cognitive factors (motivation to participate in a weight management program). Absolute weight and waist circumference will be measured with standard instruments and procedures at baseline and Week 12 by assessors blinded to arm allocation from AUBMC UHS. Intermediate self-reported measures of weight will be based on weekly check-ins, automatically prompted by the mobile apps, and collected through Web-based forms at Weeks 4 and 8.

Activity information will be captured through the phones, which will be integrated with *Google Health Kit, Apple Health*, or Samsung S Health. Participants will be asked to read and report average weekly measures of steps walked and weekly active time at Weeks 4, 8, and 12. Lark automatically tracks the amount of active time per day, whereas MyFitnessPal can estimate the number of steps walked using the phone accelerometer or external, third-party devices such as Fitbit, Misfit, and Apple Watch. As not everyone may have their phone with them all the time, we will assess physical activity also through the International Physical Activity Questionnaire, short form (IPAQ-S) [57]. IPAQ-S is one of the most widely adopted self-reported instruments to assess physical activity, which has been designed to be easily used in many languages and countries [58]. Despite the limitations of self-reported instruments compared with objective methods for measuring physical activity (eg, the “gold standard” doubly labeled water), the IPAQ-S is a feasible, easy-to-use instrument to capture physical activity [58]. As there was no budget for more reliable physical activity assessment instruments (eg, Actigraph accelerometers or pedometers), we chose the IPAQ-S because of its ease of use, as it is perceived as less daunting than the long form showing similar reliability and validity estimates [59]. Furthermore, the IPAQ-S tool has been previously used in epidemiological studies in Lebanon [60,61]. The IPAQ-S requires respondents to estimate how much time they spent while doing activities in the previous week in 4 domains: vigorous physical activity, moderate physical activity, walking, and sitting. A total physical activity score is calculated by summing the time spent in each domain. The total physical activity score and subdomain scores can be expressed in hours/week, or converted to metabolic equivalents (METs), following the IPAQ scoring protocol [62]. MET values were derived from the IPAQ Reliability Study [59], and an average MET score will be derived for each type of activity using the compendium of Ainsworth et al [63]: 1 MET equals the energy expenditure of sitting down quietly, 3.5 ml O_2_/kg/min. Physical activity will be assessed through the IPAQ-S questionnaire at baseline and Week 12.

Dietary intake data will be collected and analyzed using the Automated Self-Administered 24-hour (ASA24) Dietary Assessment Tool, version 2016, developed by the National Cancer Institute, Bethesda, MD. ASA24-2016 is based on the Automated Multiple-Pass Method, developed by the United States Department of Agriculture [64]. The multiple-pass approach in 24-hour recall provides a detailed assessment of dietary intake over the past 24 hours, including food, drinks, and supplements, as well as timing, form, portion size, the way food has been prepared, and the consumption of additions such as sugar, cream, and dressing, in addition to the source/brand of food. The 2016 version of the system includes also pictures of portions that are deemed to reduce overestimation or underestimation of food intake [65]. Since its release date (April 2016), ASA24-2016 has been used in 882 studies and 37,090 recalls have been completed. Various versions of ASA24 have also been used in self-directed weight loss interventions [66-68]. The automated version of ASA24 generally showed good reliability compared with the interviewer-administered version [69] or with the measures of true intake [70]. The ASA24-2011 version has been validated against and shown close agreement with interview-administered 24-hour dietary recalls among adults and children [70,71]. In this study, participants will complete an ASA24 at baseline and at the end of the intervention. The ASA24-2016 will be used on 3 nonconsecutive days (2 in a week day and 1 in a weekend, eg, Mondays, Thursdays, and Saturdays). Participants will access the Web-based ASA24 platform where they will be asked to recall food, drinks, or supplements they consumed in the last 24 hours. The questionnaire can be accessed via mobile phones, as it uses a responsive Web interface. Energy and macronutrients estimates will be computed using the Nutritionist Pro Software, using the United States Department of Agriculture (USDA) database (version 5.1.0, 2014, First Data Bank, Nutritionist Pro, Axxya Systems, San Bruno, CA). For composite dishes or items that are not included in the USDA database, traditional recipes will be added to the Nutritionist Pro Software, using single food items. ASA24-2016 will be assessed at baseline and Week 12. Calorie intake using the apps will be prompted at Weeks 4, 8, and 12.

Motivation to participate in a weight management program is one of the key elements of its success [72]. This construct will be assessed using the Treatment Self-Regulation Questionnaire (TSRQ) [72-74]. TSRQ includes autonomous and controlled regulation subscales. Motivation to participate in the program will be assessed at baseline and Weeks 4, 8, and 12. Additionally, stages and processes of change in weight management will be assessed using the S- and P-Weight scales [75,76]. The S- and P-Weight scales assess the cognitive predictors of weight change [77]. These scales are based on the transtheoretical model and include stages of change (S-Weight) and processes of change (P-Weight) components to assess the motivation to lose weight, which can be considered both a moderator and a mediator or covariate factor in achieving the main outcome (ie, weight loss). Stages and processes of change will be assessed at baseline and Week 12.

### Data Collection and Management

Except for the 24-hour recall/record done through the Web-based ASA24 platform, process and outcome measurements will be collected through REDCap [44], which is a secure, the Health Insurance Portability and Accountability Act compliant, Web-based survey application hosted on AUB servers. REDCap is an ideal program for managing longitudinal studies with multiple assessments.

### Sample Size

Traditional sample size calculations are not typically undertaken in nonprobability sampling and in pilot interventional studies, as these are intended to test acceptability and evaluate the process as well as to inform power calculations for subsequent studies [78]. Nevertheless, researchers recommend that sample size justifications are provided also in pilot studies [78]. In the absence of similar studies in Lebanon and in the region, it is difficult to estimate precise effect sizes. However, estimates can be derived from the average effect sizes reported in similar mobile-based weight loss interventions that had minimal researcher interaction [79-81], included in Schippers et al’s meta-analysis [21]. In these studies, the effect sizes (SMD, standardized mean difference) based on weight change ranged from *d*=0.33 [79] to 0.37 [80]. According to Whitehead et al’s tables (for SMD=0.3 and 0.4, respectively) [78], a two-arm trial, designed with 90% power and two-sided 5% significance, will require between 305 and 181 participants per arm; a pilot study will require, respectively, 45 and 33 participants per arm [78].

### Data Analyses

Descriptive statistics will be conducted for the overall characteristics of the study population through presenting the numbers and percentages for nominal or categorical variables and means and standard deviations for continuous ones. For variables assessed using multiple items (eg, uMARS scales), Cronbach alpha and corrected item-total correlations coefficients will be used to assess the internal consistency of the measured constructs, before aggregating the information (ie, averaging). Bivariate correlations and chi-square tests will be used to explore associations among demographic and psychographic variables and main study outcomes.

Missing data are expected to be minimal for most variables. Depending on the number of participants, the number of missing data points, and the distribution of the outcome variables, we will decide which missing data strategy to use (eg, full-information maximum likelihood or multiple imputation) [82]. The missing data bias will be assessed by computing a binary variable reflecting the presence or absence of missing data for each variable in the model, and then, this binary variable will be correlated with all other variables in the model as well as an array of demographic variables.

Reliability of the uMARS scales will be evaluated using both indices of interrater agreement (IRA) and interrater reliability (IRR) [83-85]. Following the recommendations from the literature [86,87], IRA will also be measured according to Brown and Hauenstein’s aWG index [88], and the adjusted average deviation index ADM (adj) [89]. IRR indices will be based on intraclass correlation coefficients measuring test-retest reliability [90,91]. Similarly, test-retest reliability estimates will be used with the ASA24 Dietary Assessment Tool and the IPAQ-S [57].

Independent samples *t* tests, one-way analysis of variances (or nonparametric alternatives where appropriate) will be used to test differences in the relevant outcome variables (behavioral, cognitive, and weight-related data) between intervention groups and between pre- and posttest. Behavioral and weight-related data will be presented using appropriate confidence intervals as suggested by Lee and colleagues [92]. The estimation of preliminary efficacy will be based on the observed trends on data over time rather than traditional inferential statistics [93]. Subgroup analyses will also include intent-to-treat versus randomized analyses to detect whether differences in the outcomes are associated with adherence to the trial (per protocol).

## Results

Funding for WaznApp study has been secured from the AUB, University Research Board. Recruitment started in February and was completed by the end of March 2018; the study will be completed by the end of June 2018. Data analyses and write-up will start over the summer and be completed by the end of 2018. Any changes in the procedures related to recruitment, eligibility criteria, outcomes, and analyses will be implemented and communicated to study participants via email within 2 weeks from the modifications in the protocol. Results will be communicated in a final report for participants.

## Discussion

This study contributes to the growing evidence on mobile apps for weight management [18,19]. The findings of this project will provide information on the feasibility of using commercially available, popular mobile apps as a standalone delivery mode for self-directed interventions for weight management. Furthermore, this study will provide preliminary insights into the potential acceptability and efficacy of an app that acts as a JITAI (Lark) at promoting weight management compared with a non-JITAI, calorie-counting app (MyFitnessPal). Lark has recently been used in an observational study, involving 70 diabetic patients who lost 2.4% of their weight at baseline after about 15 weeks [48]. MyFitnessPal has been utilized in a few weight loss trials, showing some positive effects in weight reduction when employed as a supplement to telephone coaching [30], but small, no significant effects when used as a standalone tool [31,32]. We hypothesize that the use of Lark will be associated with larger, more positive changes in cognitions, behaviors, and anthropometric measures than the other apps. This hypothesis will be tested in a larger trial. In fact, this feasibility study will be used to inform a subsequent trial with the same target population. The results of the larger study may inform other studies targeting similar workplaces in Lebanon and the MENA region, and may be used as a benchmark for further investigations in other settings and with other target groups.

Anticipated limitations of this study include slow recruitment and participation rates; the use of self-reported data, including app usage and activity; and using Web-based questionnaires that might not capture real-life hurdles and potential reasons for failure. Budgetary limitations did not allow us to include more objective measures of physical activity and dietary behaviors and a comprehensive qualitative evaluation of the trial. Nonetheless, this trial will provide information about low-cost, mobile solutions to help employees self-manage their weight.

## Appendix

Multimedia Appendix 1 Peer-reviewer report.

## Abbreviations

ASA24: Automated Self-Administered 24-hour
AUB: American University of Beirut
AUBMC: American University of Beirut Medical Center
IPAQ-S: International Physical Activity Questionnaire, short form
IRA: interrater agreement
IRR: interrater reliability
JITAI: Just-in-Time Adaptive Interventions
MARS: Mobile App Rating Scale
MET: metabolic equivalent
MENA: Middle East and North Africa
mHealth: mobile health
NCD: noncommunicable disease
TSRQ: Treatment Self-Regulation Questionnaire
UHS: University Health Services
uMARS: user version of the MARS Scale
WAKpt(s): WaznApp Karma Point(s)
WHO: World Health Organization
