# Supplementary material for: A Self-Directed Mobile Intervention (WaznApp) to Promote Weight Control Among Employees at a Lebanese University: Protocol for a Feasibility Pilot Randomized Controlled Trial
Source: JMIR Res Protoc. 2018 May 16;7(5):e133. doi: 10.2196/resprot.9793 (PMC5981057; doi:10.2196/resprot.9793)
Supplement: Multimedia Appendix 1 [file resprot_v7i5e133_app1.pdf]

AUB Research Grants

Online Review System

Reviewer(s) Comments :

Reviewer	Comments
Reviewer 1	**General comments**<br>Overall, this is a good proposal that deserves to be funded - I have some concerns with methodological details, but the research question is novel and well developed.<br>**Ethical/Political considerations: Any discussion of ethical issues – if relevant? IRB review?**<br>The project will undergo IRB review.<br>My concern is with recruitment and avoidance of undue influence.<br>**Budget: Is budget about right (i.e., reasonable), given the purpose of the study? Is it itemized?**<br>Yes<br>**Comments on section "Research Question":**<br>The problem statement is very clear and builds on previous research conducted by the PI. The rationale relies on a strong theoretical model of behaviour change, as well as practicalities of uptake and usability of apps for behaviour change related to diet and physical activity.<br><br>However, the preliminary studies section ends with the justification that this has not been done in the Lebanese context and with employees, however the relevance of studying this population is not discussed; overweight/obesity in the Lebanese context is not discussed as a significant public health issue, and the selection of an employee population is justified in the significance section with no reference to the literature on workplace interventions.<br>**Comments on section "Significance/Importance":**<br>Potential publications are outlined and the significance of the project more generally is outlined.<br>**Comments on section "Feasibility":**<br>The research is feasible and the collaboration with the Wellness program guarantees access to subjects. The investigators have the relevant experience to conduct the study.<br>**Comments on section "Theoretical framework/Literature review":**<br>The research question is well framed in a thorough literature review. Preliminary research is discussed with justification for further evaluation of the applications by users. The review presents gaps in the literature that this research will address.

Comments on section "Originality/State of the art":

The state of the art is well summarised and refers to a systematic review conducted by the PI which has informed this proposal.

Comments on section "Research design":

The mixed methods approach is interesting and appropriate in order to assess research questions 1-3. However, it is unclear how potential participants will be identified for the purposive sample and how this will be free of undue influence considering investigators are also employees within the same institution. For a pilot trial that is testing feasibility, a large number of quantitative variables are collected from a small sample of people; many measures of which are standard (anthropometry, IPAQ, 24hr recall, and don't really require piloting in and of themselves). There is a concern with relying on trends in the data if the study is not powered to detect differences over time; it is possible that this will reduce the ability of the researchers to meaningfully interpret the data.
